# E2F1 Induces KIF26A Transcription and Promotes Cell Cycle Progression *via* CDK–RB–E2Fs Feedback Loop in Breast Cancer

**DOI:** 10.3389/fonc.2020.530933

**Published:** 2021-01-11

**Authors:** Jing Xu, Lei Liu, Ranran Ma, Yawen Wang, Xu Chen, Haiting Liu, Youxin Ji, Tiantian Liu, Peng Gao

**Affiliations:** ^1^Key Laboratory for Experimental Teratology of Ministry of Education, Department of Pathology, School of Basic Medical Sciences, Cheeloo College of Medicine, Shandong University, Jinan, China; ^2^Department of Pathology, Qilu Hospital, Shandong University, Jinan, China; ^3^Department of Pathology, Affiliated Qingdao Central Hospital, Qingdao University, Qingdao, China; ^4^Department of Oncology, Affiliated Qingdao Central Hospital, Qingdao University, Qingdao, China

**Keywords:** breast cancer, cell cycle progression, transcription factors, KIF26A, CDK–RB–E2Fs

## Abstract

**Objective:**

The aim of this study was to investigate the role of KIF26A in breast cancer.

**Method:**

qRT-PCR and immunohistochemistry were conducted to explore KIF26A expression and functional contribution to breast cancer development. MTS, EDU, colony formation assays, and flow cytometry analysis were conducted to assess cell proliferation characteristics and cell cycle progression. A series of 5′-flanking region deletion plasmids and mutating the binding site, with the luciferase reporter assay, were used to identify the core promotor region of KIF26A. The prediction by software and construction of the transcriptional factor plasmids were used to identify the transcriptional factor. Chromatin immunoprecipitation assay could demonstrate transcriptional factor directly binding to the KIF26A promoter. Human Genome Oligo Microarray Assay and gene ontology (GO) and pathway analyses were used to predict the downstream pathway.

**Results:**

Our results showed that in breast cancer tissues, elevated KIF26A expression was significantly correlated with lymph node metastasis. KIF26A could promote proliferation and G0/G1 phase cell cycle progression in breast cancer cells. The core promoter region of the human KIF26A gene was located upstream of the transcription start site at position −395 to −385. The transcriptional factor E2F1 was shown to activate KIF26A expression. Furthermore, KIF26A was shown to inhibit the expression of p21, then activate CDK–RB–E2Fs pathway. The elevated E2F1 can activate the cell cycle progression and the KIF26A expression, forming feedback loop.

**Conclusions:**

The present study demonstrated that KIF26A, directly upregulated by E2F1, promoted cell proliferation and cell cycle progression *via* CDK–RB–E2Fs feedback loop in breast cancer.

## Introduction

Breast cancer (BC) is the most prevalent cancer and the leading cause of cancer-associated mortalities among women worldwide (1). Therefore, it is important to find out the molecular mechanisms of BC progression in order to facilitate the discovery of new targeted therapies.

Microarray-based gene expression profiling is a high-throughput platform that is widely applied in cancer research (2). In our previous study (3), we carried out a differential gene expression microarray analysis (GSE72307) of a group of metastatic tumors (MGs) and a group of non-metastatic tumors (non-MGs). The data indicated that kinesin family member 26 B (KIF26B) was one of the most upregulated genes in metastatic human gastric cancer (GC) tissues (3). We have demonstrated that KIF26B can promote GC proliferation and metastasis by activating the vascular endothelial growth factor (VEGF) signaling pathway (3). KIF26A, a homologous member of the kinesin family, was downregulated in MG in comparison to non-MG in GC as shown in the gene expression microarray analysis.

In the present study, we simultaneously assessed KIF26A and KIF26B expression in human BC tissues, and the results indicated that KIF26A expression, rather than KIF26B, was statistically higher in MG than in non-MG. We demonstrated that KIF26A could promote proliferation and G0/G1 phase cell cycle progression in breast cancer cells. The core promoter was confirmed and the transcriptional factor E2F1 was shown to activate KIF26A expression. Furthermore, KIF26A was shown to inhibit the expression of p21, then activate CDK–RB–E2Fs pathway. The elevated E2F1 can activate the cell cycle progression and the KIF26A expression, forming feedback loop. To our knowledge, dysregulation of KIF26A has never been reported in human tumors, nor has there been a documented role for KIF26A in BC.

## Material and Methods

### Patients and Breast Cancer Tissues

BC tissue samples for quantitative real time polymerase chain reaction (qRT-PCR) were obtained from 46 patients who underwent mastectomy at Qilu Hospital of Shandong University, China, between 2004 and 2016. Immunohistochemistry (IHC) was performed on paraffin-embedded tissue arrays of 199 cases of BC patients from Qingdao Central Hospital, China, between 2010 and 2011. The 199 patients were followed up regularly until December 31, 2016. Patients received no neo-adjuvant therapy before surgery.

This study was approved by the Ethics Committee of Qilu Hospital of Shandong University and Qingdao Central Hospital, and informed consents to use patients’ tissues for study and to reveal patients’ medical history to publish were obtained before submitting this manuscript.

### qRT-PCR

Total RNA was isolated using TRIzol (Life Technologies, Carlsbad, CA, USA), and reverse transcribed (RT) with a qPCR RT Kit (Toyobo, Osaka, Japan) according to the manufacturer’s instructions. We used SYBR Green Master Mix (Roche Diagnostic GmbH, Mannheim, Germany) and a Biosystems 7900HT instrument (Life Technologies, Carlsbad, CA, USA) to perform qRT-PCR. All of the primer sequences are provided in **Supplementary Table S1**.

### Immunohistochemistry

IHC with an antibody against KIF26A (1:500, Abcam, London, UK, ab150966) was performed on paraffin-embedded tissue sections and evaluated by two experienced pathologists. PBS served as a negative control. For each sample, we counted 500 cells from five randomly chosen fields. Staining intensity was scored as follows: 0 = negative, 1 = weak, 2 = moderate, and 3 = strong. The product of the percentage of positive cells and the intensity score was considered the final score. We used receiver operator characteristic (ROC) curves to demonstrate a cut-off point for KIF26A low expression and high expression, when specificity plus sensitivity obtained the maximum value. A final score of >85 was defined as high expression of KIF26A (**Supplementary Table S2**).

### Cell Lines and Cell Culture

Human BC cell lines MCF-7, MDA-MB-231, and MDA-MB-468 (purchased from ATCC) were used in this study and were routinely verified by STR authentication (Guangdong Hybririo Biotech Ltd, Guangzhou, China). MCF-7 cells were cultured in Dulbecco’s Modified Eagle Medium (DMEM) (Wisent Inc, Montreal, Canada), while the other two cell lines were cultured in L15 medium (GIBCO, Thermo Fisher Scientific) supplemented with 10% fetal calf serum (FBS) (Wisent), at 37°C in a humidified atmosphere with 95% air and 5% CO_2_.

### Cell Transient Transfection

Small interfering RNAs (siRNAs) targeting KIF26A (RiboBio, Guangzhou, China), E2F1 (Bioasia company, Jinan, China), or a non-targeting control (Si-NC) were transfected into BC cells using Lipofectamine 2000 (Invitrogen, Carlsbad, California, US) according to the manufacturer’s protocols.

### MTS Assay

To detect cell proliferation, we performed the MTS assay (5 mg/ml, Promega, Madison, WI, USA), according to the manufacturer’s protocol. At the same time every day, 20 µl MTS was added to each well of 96-well plates, containing 100 µl medium. After incubation for 2 h, the absorbance at 490 nm of each well was measured using a microplate reader.

### EDU

We conducted a Cell-Light EDU DNA Cell Proliferation assay (RiboBio, Guangzhou, China) using the manufacturer’s protocol. After incubation with EDU, cells were mixed with Apollo reaction cocktail, stained with DAPI, visualized and counted using a fluorescent microscope (Olympus, Tokyo, Japan).

### Clone Formation Assay

Then 500 transfected BC cells were seeded in 6-well plates. Two weeks later, clones were fixed with 10% formaldehyde (Beijing Solarbio science and Technology Co. Ltd) and stained with 0.1% crystal violet (Beyotime). Clones with diameters greater than 1 mm were measured and counted.

### Cell Cycle

Transfected BC cells, which had been serum-starved for 24 h ahead of transfection, were collected and fixed in 75% ethanol for 1 h at –20°C, washed and resuspended in cold PBS (pH = 7.4). RNase A (100 μg/ml) was then added to the cells for 30 min at 37°C. PI-stained cells were then analyzed by flow cytometry (MoFlo, Beckman-Coulter, Los Angeles, CA, USA).

### Apoptosis

Transfected BC cells were double stained using an Annexin V-FITC/PI Apoptosis Detection Kit (BestBio, Shanghai, China) and immediately analyzed by flow cytometry (MoFlo, Beckman-Coulter, Los Angeles, CA, USA).

### Western Blot

Cell lysates were generated, and protein concentrations were measured using the DC protein assay kit (Bio-Rad). Primary antibodies used in this study include KIF26A (1:300, Abcam, ab150966), Cyclin E1 (1:1,000, Abcam, ab133266), Cyclin D1 (1: 750, CST, 2978T), CDK2 (1:1,000, CST, 2546T), CDK4 (1: 1,000, CST, 12790T), CDK6 (1: 1,000, CST, 3136T), P21 (1:600, CST, 2946T), P53 (1:1,000, CST, 48818), pRB (1:1,000, Abcam, ab52975) and GAPDH (1;1,000, Abcam, ab181603). Image J software was used to analyze the gray value of the WB.

### Construction of Promoter Deletion Plasmids, Transcription Factors Overexpression Plasmids, and the Mutation Vector

The full length of the KIF26A plasmid was purchased from the company (Generay, Shanghai, China). We isolated the 5′-flanking region of the KIF26A gene, presumably harboring the regulatory sequences. The human KIF26A promoter sequences were amplified by PCR and subcloned into the PGL3-basic vector (Promega) using restriction sites for Hind III and Nhe1 (Primer sequences are provided in **Supplementary Table S3**).

We predicted and analyzed TFs using the software JASPAR (http://jaspar.genereg.net/) and ALGGEN (http://alggen.lsi.upc.es/) on the basis of the core promoter area. Full-length TFs (CREB, SP1, EGR1, KLF5, ELK1, TFAP2A, GCF4, and SP3) were amplified and cloned into the pcDNA3.1 expression vector in our laboratory (Primer sequences are provided in **Supplementary Table S4**). C/EBP alpha was provided by Professor Anli Jiang at Shandong University, China. E2F1 overexpression vector (Bioasia company, Jinan, China) was cloned into the P-Enter expression vector.

The E2F1 binding site sequence with the promoter region was predicted by JASPAR (http://jaspar.genereg.net/). The point mutations were synthesized (Bioasia Company, Jinan, China).

All of these plasmids were transfected with Turbofect (Thermo Fisher Scientific, Lithuania) and verified by DNA sequencing.

### Luciferase Reporter Assay

The luciferase reporter constructs, along with pRL-TK, a Renilla reniformis luciferase reporter vector, were transiently transfected into 293T and MCF-7 cells. We obtained the relative expression of firefly luciferase by normalizing to Renilla luciferase activity.

### Chromatin Immunoprecipitation

CHIP analysis was performed with the EZ CHIP kit (Merck Millipore) and CHIP+E2F1-CHIP validated antibody and primer set (Merck Millipore, 17-10061), according to the manufacturer’s instructions with some modifications. Protein-DNA complexes were immunoprecipitated following the manufacturer’s protocol. Isolated DNA was then analyzed by PCR using the following primers containing the KIF26A promoter: Forward primer 5′-CTCCTGGGAGACGGGAGAG-3′ and Reverse primer 5′-GCACTCCAAGGACCCCAAC-3′. A comparative Ct method was used to calculate the relative fold enrichment of the immunoprecipitated DNA in each sample.

### Human Gene Expression Microarray

In our research group, we transfected cancer cells with KIF26A siRNA and Si-NC, and then used the Whole Human Genome Oligo Microarray Assay (KangChen Bio-Tech, Shanghai, China) to analyze differential gene expression between the two groups. We performed gene ontology (GO) and pathway analyses using standard enrichment computation methods to find the downstream regulatory mechanism of KIF26A (http://www.kegg.jp/).

### Statistical Analysis

Experiments were performed in duplicate or triplicate and independently repeated three times. The SPSS software (Version 15.0) and GraphPad Prism 6 (GraphPad Software, Inc., San Diego, CA, USA) were used for statistical analysis. Two-tailed Fisher’s exact test was used to calculate the correlation between KIF26A expression and clinicopathological parameters. The Kaplan–Meier method and the Log-rank test were used to determine the survival. Two-tailed Student’s t test was used for other data. P <0.05 was considered to be statistically significant.

## Results

### Correlation Between KIF26A Expression and Clinicopathological Variables of Breast Cancer Patients

**Figure 1:**
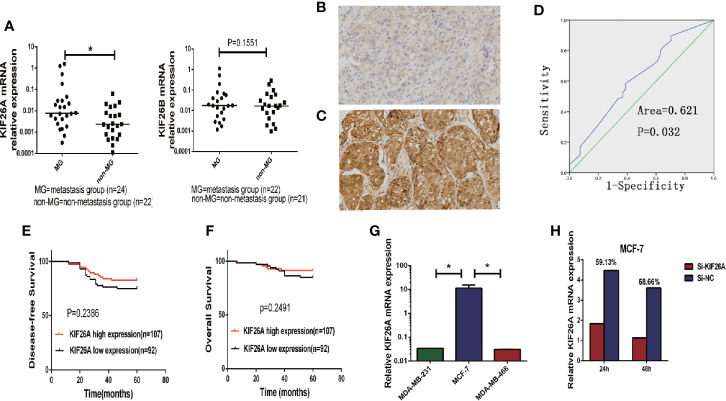
The correlation between KIF26A expression and clinicopathologic features in BC tissues. **(A)** KIF26A expression levels were statistically higher in LN MG (n = 24) than non-MG (n = 22) but KIF26B expression was not significantly correlated with LN metastasis. **(B, C)** KIF26A protein expression detected by IHC was reversely identical with tissue grade. **(D)** ROC curves confirmed that KIF26A expression could clearly separate the low or high grade cases, with a cutoff point of 85% for KIF26A IHC outcome and the AUC of 0.613. **(E, F)** KIF26A expression was proved not statistically positively correlated with 5-year disease free survival and overall survival of BC. **(G)** Analysis of endogenous KIF26A levels in three BC cell lines showed that KIF26A levels were markedly increased in low-aggressive cell lines MCF-7. **(H)** The siRNA transfection efficacy of mRNA was 59.13 and 68.66% in MCF-7 cells on 24 and 48 h, respectively. * means P < 0.05.

We analyzed the expression of KIF26A in 46 BC samples using qRT-PCR, which indicated that KIF26A was expressed at a significantly higher level in MG (n = 24) than in non-MG (n = 22) tumors (**Figure 1A**, P = 0.0468).

To further evaluate the expression of KIF26A in BCs, we performed IHC in 199 cases of paraffin-embedded BC tissues. ROC curves confirmed that KIF26A expression could clearly separate the low-grade and high-grade cases (**Figures 1B, C**), with a cutoff point of 85 for KIF26A based on IHC and an area under the curve (AUC) of 0.613 (**Figure 1D**, P=0.014). Moreover, elevated KIF26A expression was significantly correlated with positive lymph node (LN) metastasis, positive ER expression, and luminal subtype of BC (**Table 1**).

**Table 1:** Correlation between KIF26A expression and clinicopathological features.

variants	Total case (199)	KIF26A low expression	KIF26A high expression	p value
Age				0.2522
≤50 years	86	44	42	
>50 years	113	48	65	
Tissue grade				0.04
1	17	5	12	
2	124	54	70	
3	55	33	22	
Pathologic stage				1
pT1	88	41	47	
pT2-3	111	51	60	
LN status				0.0483
non-metastasis	112	57	55	
metastasis	70	25	45	
DFS				1
Yes	125	63	62	
no	25	12	13	
lost	51	17	34	
ER Status				0.043
Positive	115	46	69	
Negative	83	46	37	
PR Status				0.2567
Positive	100	42	58	
Negative	99	50	49	
Her2 Status				0.8611
Positive	41	18	23	
Negative	156	72	84	
Ki-67 expression (%)				0.0187
≤20	88	33	55	
>20	99	55	44	
Molecular subtype				
Luminal type	118	47	71	0.0301
non-luminal type	79	44	35	

However, KIF26A expression was not significantly correlated with age, pathologic stage, 5-year disease free survival, and overall survival of BC (**Figures 1E, F**, log rank test, P > 0.05).

### KIF26A Promoted Proliferation and Cell Cycle Progression in Breast Cancer Cells *In Vitro*

Next, the functional role of KIF26A in BC was investigated. Analysis of endogenous KIF26A levels in three BC cell lines showed that KIF26A levels were high in the less aggressive cell line MCF-7, but low in the highly aggressive cell lines MDA-MB-231 and MDA-MB-468 (**Figure 1G**). The siRNA transfection efficacy of mRNA was 59.13 and 68.66% in MCF-7 cells at 24 and 48 h, respectively (**Figure 1 H**).

**Figure 2:**
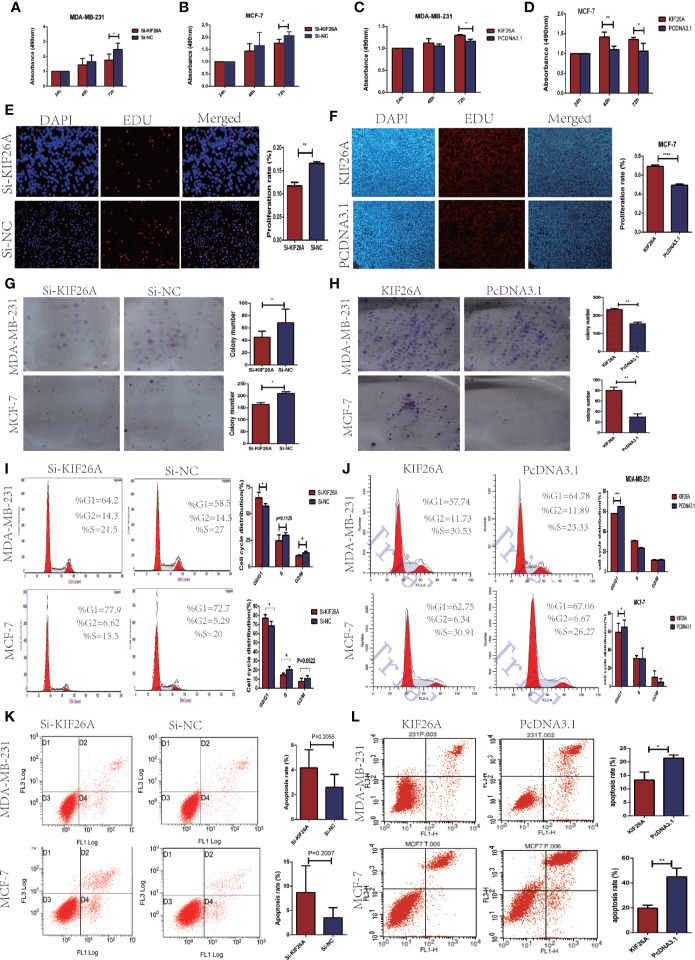
KIF26A promote the effect of proliferation and cell cycle progression on BC cells *in vitro*. **(A–D)** Results from MTS assay showed that BCs exhibited cell proliferation transfected with KIF26A siRNA **(A, B)**, and promote cell proliferation with KIF26A overexpression **(C, D)**. **(E, F)** EDU assays confirmed the promotion effect of KIF26A on cell proliferation in BCs with KIF26A knockdown and overexpression. **(G, H)** In the soft agar of the colony formation assay, the colonies were smaller and fewer in KIF26A-knockdown BC cells, and larger in KIF26A overexpression cells, compared with the control. **(I, J)** KIF26A knockdown induced G0/G1 phase cell cycle arrest compared with that of Si-NC in BCs with flow cytometry detection, and the opposite effect on the KIF26A overexpression group. **(K, L)** KIF26A knockdown could promote cell apoptosis in BCs, and overexpression group got the opposite effect. * means P < 0.05. ** means P < 0.01. **** menas P < 0.0001.

MTS assay showed that BC cells transfected with KIF26A siRNA exhibited lower cell proliferation, in comparison to control cells transfected with Si-NC (**Figures 2A, B**). EDU assays confirmed the inhibitory effect of KIF26A knockdown on cell proliferation in BC cells (p =0.0052) (**Figure 2E**). Colony formation assay was also performed to further confirm the inhibitory effect of KIF26A knockdown on the ability of BC cells to form colonies (**Figure 2G**). In soft agar assays, KIF26A knockdown cells generated fewer and smaller colonies in comparison to control Si-NC cells. These data demonstrated that KIF26A knockdown was involved in the inhibitory role of tumor growth of BC cells *in vitro*. On the contrary, KIF26A overexpression effectively promotes the cell proliferation (**Figures 2C, D, F, H**).

Subsequently, flow cytometry was performed to analyze the effect of KIF26A on cell cycle progression. As shown in **Figure 2I**, KIF26A knockdown induced G0/G1 phase cell cycle arrest in comparison to the Si-NC group, whereas KIF26A overexpression promotes the transition from G1 to S phases of the cell cycle (**Figure 2J**). Annexin V-FITC/PI apoptosis assay was performed to investigate the role of KIF26A in cell apoptosis. KIF26A knockdown did not significantly affect cell apoptosis in BC cells (p > 0.05) (**Figure 2K**), but KIF26A overexpression inhibits the cell apoptosis significantly (**Figure 2L**). These results indicated that KIF26A promoted proliferation and cell cycle progression in BC cells.

### Identification of the Core Promoter Region of KIF26A

Next, we explored the mechanism of KIF26A up-regulation. Transcription regulators can bind to the promoters of genes and activate their expression. Thus, the promoter of KIF26A and its transcription activator were identified.

**Figure 3:**
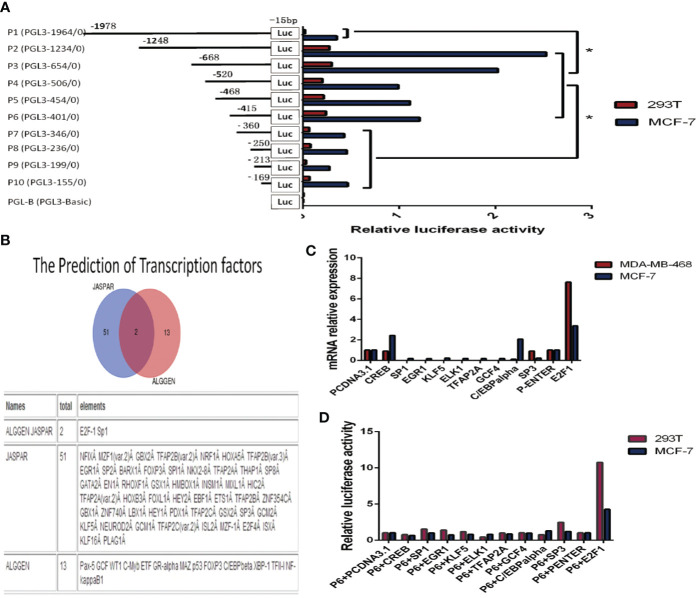
Cloning of the KIF26A promoter and verification of the transcription factors (TFs). **(A)** Various fragments of −1978/−15 TSS were cloned, named P1 to P10. Deletions from P6 to P7 significantly decreased the luciferase activity. **(B)** KIF26A gene promoter contains various TF binding sites, including E2F1 and SP1 as the intersection elements. **(C)** KIF26A mRNA expression was higher in E2F1-overexpressing cells. **(D)** Luciferase assays suggested that E2F1, rather than other nine transcription factors and empty vector, and P6 co-transfection could much more remarkably enhance the luciferase activity in 293T and MCF-7 cells lines. **Figure 4**: E2F1 was verified to transcriptionally activate KIF26A, and the core promoter region was further identified. * means P < 0.05.

We cloned the DNA sequence (–1978/–15 relative to the transcription start site (TSS) of the KIF26A gene promoter region into the promoter-less pGL3-Basic luciferase reporter plasmid, and named the plasmid P1 (pGL3-1964). Similarly, reporter plasmids containing shorter fragments of the KIF26A promoter region were constructed and named P2 (pGL3-1234, containing sequence –1248/–15), P3 (pGL3-654, containing sequence –668/–15), P4 (pGL3-506, containing sequence –520/–15), P5 (pGL3-454, containing sequence –468/–15), P6 (pGL3-401, containing sequence –415/–15), P7 (pGL3-346, containing sequence –360/15), P8 (pGL3-236, containing sequence –250/–15), P9 (pGL3-199, containing sequence –213/–15), and P10 (pGL3-155, containing sequence –169/–15) (**Figure 3A**).

The plasmids were co-transfected with pRL-TK plasmids into MCF-7 and 293T cells, and luciferase assays were performed to detect the promoter activity of KIF26A. As shown in **Figure 3A**, deletion of sequences between –415 and –360 significantly decreased luciferase activity, indicating that the promoter region spanning these 55 bp nucleotides were required for basal transcription activity of the KIF26A gene.

### E2F1 Was Shown to Activate the KIF26A Promoter and the Core Promoter Region Was Further Identified by Mutational Analysis

The 55 bp nucleotide sequence from –415 to –360 was subjected to computer analysis to identify conserved potential TFs binding sites. The software JASPAR and ALGGEN predicted that the KIF26A gene promoter region contained 51 and 13 putative TF binding sites, respectively, including E2F1 and SP1 as the intersection elements (**Figure 3B**).

Concerned with the score of the possible TFs predicted by JASPAR, we overexpressed CREB, SP1, EGR1, KLF5, ELK1, TFAP2A, GCF4, C/EBP alpha, SP3, and E2F1 by transfecting overexpression vector, pcDNA3.1 and P-Enter as empty vectors in BC cells. By qRT–PCR, the results showed that KIF26A mRNA expression was significantly higher in E2F1-overexpressing cells in comparison to cells overexpressing the other nine TFs and the empty vector (**Figure 3C**). The results of the luciferase assays demonstrated that E2F1, rather than the other nine TFs and the empty vector, and P6 co-transfection could remarkably enhance the luciferase activity in 293T and MCF-7 cell lines (**Figure 3D**). Therefore, E2F1 was identified to be involved in the activation of KIF26A.

**Figure 4:**
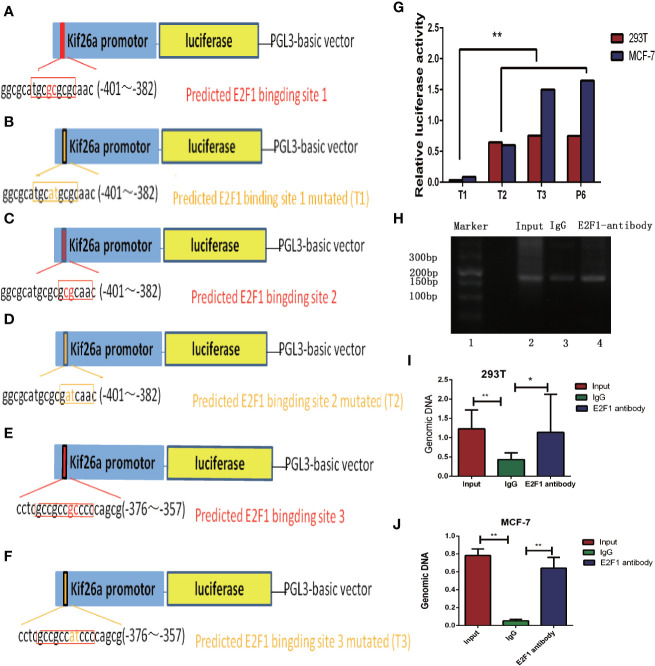
E2F1 was verified to be involved in the activation of the KIF26A through directly binding to the KIF26A promotor area and the core promoter region was further identified by mutation vector. **(A, C, E)** The predicted E2F1 binding site with the promotor area include binding site 1 (−395/−385), binding site 2 (−390/−383) and binding site 3 (−372/−362). **(B, D, F)** We constructed mutational PGL3-basic vectors by replace two bases of E2F1 binding site accordingly, named as T1, T2 and T3. **(G)** The activity of the luciferase reporter gene that carries T1 was inhibited significantly obvious than T2, T3 and P6 in 293T and MCF-7 cells, indicating the core promotor area located within the −395 to −385 region relative to the Tss. **(H–J)** The products of chromatin immunoprecipitation (CHIP) assay demonstrated by agarose gel electrophoresis **(H)** and the RT-qPCR outcome **(I, J)** indicated that the sequence binding to the antibodies precipitated proteins were proved to include the KIF26A promoter region by E2F1 antibody and the input(positive control antibody), but not by non-specific IgG (negative control antibody). * means P < 0.05. ** means P < 0.01.

The predicted E2F1 binding site within the promoter region included binding site 1 (–395/–385), binding site 2 (–390/–383), and binding site 3 (–372/–362) (**Figures 4A, C, E**) We constructed mutated PGL3-basic vectors by replacing two bases in the E2F1 binding site and named these mutated vectors T1, T2, and T3 (**Figures 4B, D, F**) We transfected 293T and MCF-7 cells with T1, T2, or T3 in combination with P6. The activity of the luciferase reporter gene that carried T1 was more significantly inhibited than the genes carrying T2 or T3 (P value <0.01) (**Figure 4G**). Therefore, we determined that the predicted E2F1 binding site 1 (–395/–385) is the core promoter region of KIF26A.

### E2F1 Could Directly Bind to the KIF26A Promoter Region and Promote KIF26A Expression

To determine whether E2F1 could directly bind to the KIF26A promoter region, we performed CHIP experiments in 293T and MCF-7 cells. E2F1-associated DNA fragments were immunoprecipitated using an anti-E2F1 antibody. Normal rabbit IgG was used as a negative control and input as a positive control. DNA fragments were then amplified by PCR and qRT-PCR using primers surrounding the predicted E2F1 binding site 1 (–395/–385).

The PCR products separated by agarose gel electrophoresis (**Figure 4H**), and qRT-PCR analysis (**Figures 4I, J**) indicated that the DNA sequence of the KIF26A promoter region was bound to the precipitated protein eluted by the E2F1 antibody, but not by the non-specific IgG (negative control antibody). These data demonstrated that the minimal promoter of human KIF26A was located within the –395 to –385 region relative to the TSS, which was directly bound by the transcription factor E2F1.

**Figure 5:**
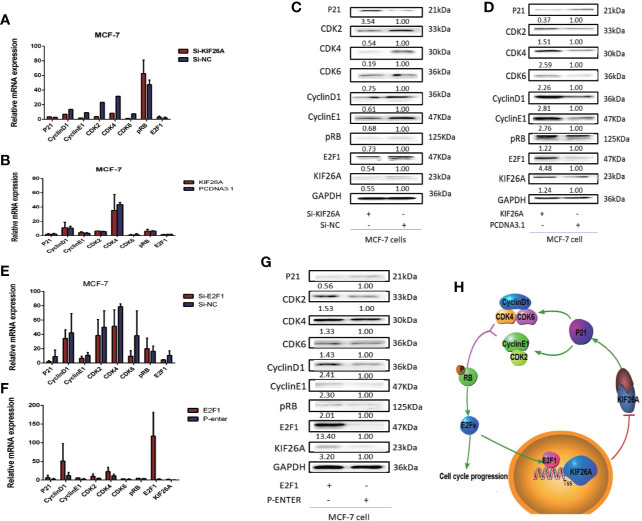
KIF26A could modulate the expression of the P21 pathway. **(A–D)** P21 pathway was influenced significantly at the protein level, not mRNA level. **(E–G)** The elevated E2F1 activated the KIF26A expression and inhibited p21 pathway. **(H)** Mode pattern: KIF26A, directly upregulated by E2F1, promoted cell proliferation and cell cycle progression *via* CDK–RB–E2Fs feedback loop in BC.

As E2F1 may have a role in the transcription regulation of KIF26A, we further investigated the effect of E2F1 overexpression or knockdown on KIF26A expression. WB results showed that overexpressing E2F1 increased, while E2F1 knockdown decreased the expression of KIF26A expression by RT-qPCR and WB (**Figures 5E–G**). The data suggested that E2F1 promoted KIF26A expression in BC cells.

### E2F1 Could Activate the KIF26A Expression and Inhibit the Expression of p21, Then Activate CDK–RB–E2Fs Pathway, Forming Feedback Loop

To further confirm how KIF26A regulated proliferation and cell cycle progression, we performed gene ontology (GO) and pathway analyses using the standard enrichment computation method of a comparative analysis of gene expression profiles of KIF26A siRNA and Si-NC cells, which indicated that the p21 pathway may play a role in regulating the cell cycle.

P21 pathway markers were not significantly increased at the mRNA levels in the KIF26A knockdown or overexpression BC cells (**Figures 5A, B**). However, the expression of P21 was significantly elevated, and CDK2/4/6, Cyclin D1/E1, pRB and E2F1 was depressed in the KIF26A knockdown group (**Figure 5C**), and opposite expression in the KIF26A overexpression group in WB (**Figure 5D**), which indicated that KIF26A mainly affected the p21 pathway at the post-transcription level.

KIF26A was shown to inhibit the expression of p21, then activated CDK–RB–E2Fs pathway. The elevated E2F1 transcription factors can activate the cell cycle progression, and also activated the KIF26A expression and inhibited the expression of p21, then activate CDK–RB–E2Fs pathway, verified by RT-qPCR (**Figures 5E, F**) and WB (**Figure 5G**), forming feedback loop (**Figure 5H**).

## Discussion

BC is the most common malignant tumor in women worldwide. Excessive growth and metastasis are the two main phenotypes of malignant tumors. The molecular pathogenesis behind the overgrowth and metastases of BC is an area of intense research.

Based on the results of our differential gene expression microarray analysis of MG and non-MG (GSE72307), we investigated one of the most upregulated genes in MG, KIF26B, and demonstrated that KIF26B could promote GC proliferation and metastasis by activating the vascular endothelial growth factor (VEGF) signaling pathway (3). However, the role of KIF26A, a homologous family member of KIF26B, in cancer remains unknown. Our study with BC tissues demonstrated that KIF26A expression was statistically higher in MG than in non-MG, and correlated with positive LN metastases. These results indicated that KIF26A may be a potential prognostic marker in BC. Therefore, we explored the functional role of KIF26A in BC.

Kinesin super family proteins (KIFs) are involved in many biological and physiological processes, including microtubule-dependent molecular motors, microtubule stabilizers, and depolymerizers, which are fundamental to cellular morphogenesis and mammalian development (4). KIF26A, located at chromosome 14q32.33, is a member of the Kinesin-11 family (5). Unlike other KIFs, KIF26A has an atypical motor domain that can bind to microtubules but does not hydrolyze ATP (6). Zhou et al. found that KIF26A worked as a microtubule-stabilizing factor in enteric neurons by directly binding and inhibiting Grb2/SHC complex formation, thereby disrupting Ret tyrosine kinase activation and subsequent signaling of ERK1/2 and phosphatidylinositol-3 kinase (PI3K) pathways (6). Caso et al. demonstrated that KIF26A was associated with anterior cruciate ligament non-contact injury (7).

Our investigation revealed that KIF26A promoted BC cell proliferation, as demonstrated by MTS, EDU, and colony formation assays. Flow cytometry analysis also revealed that KIF26A induced G0/G1 phase cell cycle progression and inhibited cell apoptosis in BCs. These results suggested that KIF26A could promote cell proliferation and cell cycle progression.

Normal cell function and tissue homeostasis is maintained by a balance between proliferation and apoptosis (8). Cancer is a disease in which clones of malignant cells escape this balance and proliferate inappropriately without compensatory apoptosis (9–11).The success of cancer therapies greatly relies on the extent to which they can induce tumor cell death while allowing survival of normal tissue. Investigations into the role of cell cycle regulatory molecules in the induction of apoptosis in cancers afford us a better understanding of the biology of cancer cells (12).

Currently, neither the mechanism of KIF26A in promoting proliferation and cell cycle progression, nor its pathogenic function in BC has been uncovered.

In the present study, the 5′-flanking region of the KIF26A gene was isolated and cloned, and the promoter region was verified by luciferase reporter assay. E2F1 was predicted and shown to be involved in the activation of the KIF26A promoter. We further identified that the core promoter region of human KIF26A was located at the –395 to –385 region relative to the TSS. CHIP assay demonstrated that E2F1 directly bound the KIF26A promoter. E2F1 overexpression significantly increased the luciferase activity of the KIF26A promoter and also increased the relative mRNA expression and protein expression of KIF26A and the downstream pathway marker. Furthermore, we silenced or overexpressed E2F1 and found that KIF26A expression was significantly influenced. These results indicated that the KIF26A was a direct transcription target of E2F1.

E2F1 is a member of the E2F TF family and plays important roles in regulating G1/S transition, DNA synthesis, and apoptosis (13–16). Hallstrom et al (17). found that two categories of E2F1 target genes, related to proliferation and apoptosis, are expressed in a mutually exclusive fashion in breast and ovarian cancers. The present study indicated that KIF26A was a target gene of E2F1 to promoted cell proliferation and cell cycle progression in BC cells.

The control of cellular proliferation, including the entry from quiescence (G0) into the cell cycle (G1) and passage into the DNA replication (S) phase, is tightly regulated by cell size, mitogenic stimulation, and absence of signals that block proliferation (17). Modulation of cell cycle progression is considered to be a promising approach to control tumor growth (18).

To further confirm how KIF26A may be involved in cell proliferation and the cell cycle, we performed GO and pathway analyses using the standard enrichment computation method of a comparative analysis of the gene expression profile of KIF26A siRNA and si-NC cells, which indicated that the P21 pathway may play a role in KIF26A-regulated cell cycle. We demonstrated KIF26A could inhibit the expression of p21, then activate CDK–RB–E2F pathway. The elevated E2F transcription factors can activate the cell cycle progression and also activate the KIF26A expression, forming feedback loop.

Cyclins are proteins that control cell cycle progression. Cyclin D1/CDK4 and Cyclin E/CDK2 regulate transition to the G1 phase while Cyclin A/CDK2 regulates entry into G2/M phase (19). Cyclin D1, one of the most important cell cycle regulators, is responsible for the transition from G1 phase to S phase by forming complexes with CDK4 and CDK6 (20). We demonstrated that knockdown of KIF26A induced G0/G1 phase cell cycle arrest, which was consistent with the decreased expression of CDK2, 4, 6, Cyclin D, and Cyclin E.

P21 is known as an important cell cycle regulator with a wide range of kinase inhibitory activity, which can effectively inhibit CDK2 and cyclins to prevent cells from going through the G1/S phase checkpoint and to inhibit cell proliferation (21, 22). CDK4/6 and CDK2 bind to CyclinD and CyclinE, respectively, which promote the phosphorylation of Rb protein, thereby releasing E2F transcription factors and activating genes necessary for S phase and DNA synthesis (23). Multiple studies have demonstrated that the CDK–RB–E2Fs pathway is critical for the control of cell proliferation, and the CDK–RB–E2Fs pathway is a driver of multiple hallmarks of breast cancer and consequently could be a good target for therapy in this disease (24).

We demonstrated that E2F1 induces KIF26A transcription and promotes cell cycle progression *via* the CDK–RB–E2Fs feedback loop in breast cancer. KIF26A was identified to be a novel biomarker for cell cycle progression in BC and can lead to the development of novel targeted therapies.

## Data Availability Statement

The datasets generated for this study are available on request to the corresponding authors.

## Ethics Statement

This study was approved by the Ethics Committee of Qilu Hospital of Shandong University and the affiliated Qingdao Central Hospital of Qingdao University. The patients/participants provided their written informed consent to participate in this study.

## Author Contributions

PG and TL designed the research. JX and LL conducted the experiments. RM, YW, XC, and HL guided the experiments and data analysis. JX drafted the manuscript. PG and YJ revised the manuscript. All authors contributed to the article and approved the submitted version.

## Funding

This study was supported by the National Natural Science Foundation of China (Grant Nos. 81872362 and 81672842) and the Taishan Scholars Program of Shandong Province (Grant No. ts201511096) and the National Key R&D Program of China (No. 2017YFC1308600).

## Conflict of Interest

The authors declare that the research was conducted in the absence of any commercial or financial relationships that could be construed as a potential conflict of interest.
