# Supplementary material for: E2F1 Induces KIF26A Transcription and Promotes Cell Cycle Progression via CDK–RB–E2Fs Feedback Loop in Breast Cancer
Source: Front Oncol. 2021 Jan 11;10:530933. doi: 10.3389/fonc.2020.530933 (PMC7832431; doi:10.3389/fonc.2020.530933)
Supplement: Supplementary file 1 [file Table_1.docx]

| **Table S1** primers for qRT-PCR | |
| --- | --- |
| names of the primer | Primer sequence (5'-3': sense+ antisense) |
| KIF26A | F(sense): TCAGAGAGGAGACGGAGTGTG R(antisense): AAGTCCAAATCAAGGTCAAAGGC |
| CCND1 | F(sense): CAATGACCCCGCACGATTTC R(antisense): CAATGACCCCGCACGATTTC |
| CyclinE1 | F(sense): AGAGGAAGGCAAACGTGACC R(antisense): TTGTCAGGTGTGGGGATCAG |
| CDK2 | F(sense): GACACGCTGCTGGATGTCA R(antisense): GGAATGCCAGTGAGAGCAGA |
| CDK4 | F(sense): CCTCTCTAGCTTGCGGCCT R(antisense): GTTCCTACGGCCCCATACA |
| CDK6 | F(sense): TCACACCGAGTAGTGCATCG R(antisense): CGTGACGACCACTGAGGTTA |
| P21 | F(sense): GCAGACCAGCATGACAGATTTC R(antisense): ATGTAGAGCGGGCCTTTGAG |
| P53 | F(sense): TAGTGTGGTGGTGCCCTATG R(antisense): CCAGTGTGATGATGGTGAGG |
| E2F1 | F(sense): CCGTGGACTCTTCGGAGAAC R(antisense): ATCCCACCTACGGTCTCCTC |
| pRB | F(sense): AGGACCGAGAAGGACCAACTGATC  R(antisense): CTGGAAGGCTGAGGTTGCTTGTG |
