# Supplementary material for: E2F1 Induces KIF26A Transcription and Promotes Cell Cycle Progression via CDK–RB–E2Fs Feedback Loop in Breast Cancer
Source: Front Oncol. 2021 Jan 11;10:530933. doi: 10.3389/fonc.2020.530933 (PMC7832431; doi:10.3389/fonc.2020.530933)
Supplement: Supplementary file 3 [file Table_3.docx]

| **Table S3** Primer sequences for construction of KIF26A promoter reporter plasmids | |
| --- | --- |
| Names of plasmids | Primer sequence (5'-3'): sense+ antisense |
| P1 (PGL3-1978/-15) | F1(sense): CCCAAGCTTCCGCACTTTCCCCCATTAGC +R1(antisense): CTAGCTAGCAAGAGCCCGGCAGGCGCTCC |
| P2 (PGL3-1248/-15) | F2: CCCAAGCTTGTGGGCAAAACFFAAAGGGT +R1 |
| P3 (PGL3-668/-15) | F3: CCCAAGCTTACACCGCGGCGACACTAACCC +R1 |
| P4 (PGL3-520/-15) | F4: CCCAAGCTTGCAAGAGCGTCCCCTCCGCAG +R1 |
| P5 (PGL3-468/-15) | F5: CCCAAGCTTGTCGGCGCCTCCTGGGAGAC +R1 |
| P6 (PGL3-415/-15) | F6: CCCAAGCTTGGCGCATGCGCGCGCGCAAC +R1 |
| P7 (PGL3-360/-15) | F7: CGCAAGCTTGGGCAGGGACTCACCTAACAG +R1 |
| P8 (PGL3-250/-15) | F8: CCCAAGCTTACTCTCTGGGCTCCCGCCCCT +R1 |
| P9 (PGL3-213/-15) | F9: CCCAAGCTTAGCCCGAGCCCCGCCCTTC +R1 |
| P10 (PGL3-169/-15) | F10: CCCAAGCTTCGCGCCCACCGGAGCCACC +R1 |
