# Supplementary material for: E2F1 Induces KIF26A Transcription and Promotes Cell Cycle Progression via CDK–RB–E2Fs Feedback Loop in Breast Cancer
Source: Front Oncol. 2021 Jan 11;10:530933. doi: 10.3389/fonc.2020.530933 (PMC7832431; doi:10.3389/fonc.2020.530933)
Supplement: Supplementary file 4 [file Table_4.docx]

| **Table S4** Primer sequence for transcription factors construction of overexpression plasmids | |
| --- | --- |
| names of the primer | Primer sequence (5'-3'): sense+ antisense |
| CREB | F(sense): CTC AAGCTT ATGACCATGGAATCTG R(antisense): GCGTCTAGA TTAATCTGATTTGTGGC |
| SP1 | F(sense): GTCAAGCTTATGAGCGACCAAGATCACT R(antisense): GTCTCTAGA TCAGAAGCCATTGCCAC |
| EGR1 | F(sense): CCCAAGCTTAGCTCTCCAGCCTGCTCGT R(antisense): CACTCTAGATTTCCCCTTTCCCTTTAGCAA |
| KLF5 | F(sense): CAGAAGCTTATGGCTACAAGGGTGCTGA R(antisense): GCGGAATTCTCAGTTCTGGTGCCTCTTC |
| ELK1 | F(sense): CACAAGCTTCACTCCCCCAGCGATGGAC R(antisense): CGCTCTAGATGGTAGTAGTCATGGCTTCTG |
| TFAP2A | F(sense): GGCAAGCTTATGCTTTGGAAATTGACGG R(antisense): GTCTCTAGATCACTTTCTGTGCTTCTCC |
| GCF4 | F(sense): GTAGGTACCCCATGGCTCACAGGCCGAA R(antisense): GATCTCGAGGGCACCAGCTTCTTCTCAAACA |
| SP3 | F(sense): CTCAAGCTTATGACCGCTCCCGAAAAGCCC  R(antisense): CCGAATTCTTACTCCATTGTCTCATTTCCAGAA |
